# The Periconceptional Environment and Cardiovascular Disease: Does *In Vitro* Embryo Culture and Transfer Influence Cardiovascular Development and Health?

**DOI:** 10.3390/nu7031378

**Published:** 2015-02-18

**Authors:** Monalisa Padhee, Song Zhang, Shervi Lie, Kimberley C. Wang, Kimberley J. Botting, I. Caroline McMillen, Severence M. MacLaughlin, Janna L. Morrison

**Affiliations:** Early Origins of Adult Health Research Group, School of Pharmacy and Medical Sciences, Sansom Institute for Health Research, University of South Australia, Adelaide, SA 5001, Australia; E-Mails: padmy002@mymail.unisa.edu.au (M.P.); song.zhang@unisa.edu.au (S.Z.); shervi.lie@mymail.unisa.edu.au (S.L.); kimberley.wang@mymail.unisa.edu.au (K.C.W.); kb555@cam.ac.uk (K.J.B.); caroline.mcmillen@newcastle.edu.au (I.C.M.); severence.maclaughlin@gmail.com (S.M.M.)

**Keywords:** assisted reproductive technology, *in vitro* fertilization, periconceptional period, cardiovascular disease, epigenetics

## Abstract

Assisted Reproductive Technologies (ARTs) have revolutionised reproductive medicine; however, reports assessing the effects of ARTs have raised concerns about the immediate and long-term health outcomes of the children conceived through ARTs. ARTs include manipulations during the periconceptional period, which coincides with an environmentally sensitive period of gamete/embryo development and as such may alter cardiovascular development and health of the offspring in postnatal life. In order to identify the association between ARTs and cardiovascular health outcomes, it is important to understand the events that occur during the periconceptional period and how they are affected by procedures involved in ARTs. This review will highlight the emerging evidence implicating adverse cardiovascular outcomes before and after birth in offspring conceived through ARTs in both human and animal studies. In addition, it will identify the potential underlying causes and molecular mechanisms responsible for the congenital and adult cardiovascular dysfunctions in offspring whom were conceived through ARTs.

## 1. Introduction

In recent years, the use of Assisted Reproductive Technologies (ARTs) have increased rapidly as a result of increasing infertility rates in humans and increasing demand for the reproduction of livestock with desired genetic characteristics. There are, however, a range of controversial issues surrounding *in vitro* embryo culture and embryo transfer, both of which are important processes in ARTs. These processes occur during the periconceptional period and are known to involve manipulation of the nutritional environment. Hence, to understand the link between ARTs and their effects on cardiovascular health, we need to understand and address these major questions:

(1) When is the periconceptional period?
(2) Why is the periconceptional period a critical window of embryonic development?
(3) What are the different procedures involved in ARTs and how do these impact the periconceptional environment?
(4) What is the evidence that the periconceptional environment influences cardiovascular health in fetal life and in adulthood?
(5) What is the evidence that ARTs influence cardiovascular health before and after birth?
(6) What are the most likely mechanisms linking ARTs and risk of cardiovascular disease in fetal and adult life?

## 2. When Is the Periconceptional Period?

**Figure 1:**
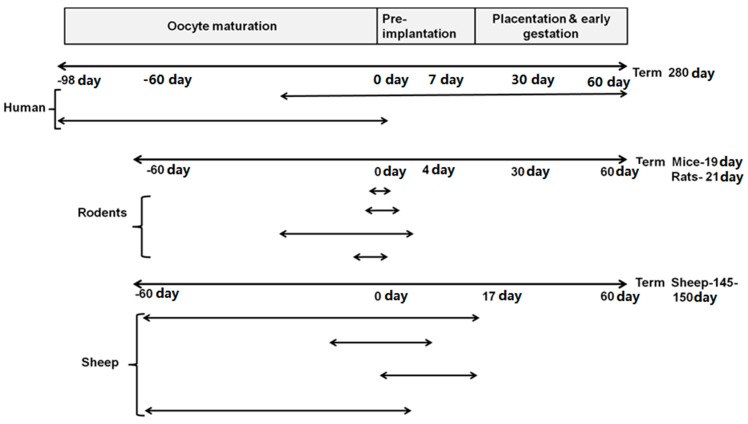
Different models of the periconceptional period in studies in humans, rodents and ruminants include different stages of oocyte and embryo development. Note: Implantation occurs at different days after conception across species (Human, 7–9 days; Rodents, 6–7 days; Sheep 16 days) [4,5,6,7,8,9,10,13,16,17].

ARTs involve manipulations that occur during oocyte maturation, fertilization and preimplantation, each of which is part of the periconceptional period and are likely to involve changes in the nutritional environment during this period [1,2].The term “periconceptional” is defined as the period before and immediately after the time of conception and is a critical period during early development [3]. Most human studies have included different time frames in defining the periconceptional period and these depend on the specific research questions (Figure 1). For example, a study of maternal multivitamin supplementation in the periconceptional period on congenital abnormalities, included from one month prior to conception to about two months after conception [4]. Another study investigating the effect of periconceptional maternal characteristics on embryonic development has defined 14 weeks prior to conception as the periconceptional period [5].

Similar to human studies, animal studies have used different timings around conception to define the periconceptional period (Figure 1). For example, the periconceptional period is defined as 3–6 days before and 1 day after conception in one study and 3.5 days before and after conception in another study in mice (term, 19 days) [6,7]. In rats (term, 21 days), the periconceptional period is defined as 3 weeks prior to and 5 days after conception, whereas in another study, it is defined as 4.25 days after conception [8,9]. In sheep (term, 145–150 days), different definitions of the periconceptional period have been used to investigate the impact of maternal nutrition in the periconceptional period on the development and health of the offspring (Figure 1). The Auckland model defines the periconceptional period as extending from 60 days prior to conception until 30 days gestation (term, 145 days) [10,11]. This period includes oocyte maturation, preimplantation and postimplantation of the embryo as well as early placentation [12], because implantation occurs on day 16 of gestation in sheep [12]. The Nottingham model includes the period between day 0 till day 30 of gestation (term, 147 days), which covers embryo/blastocyst development, preimplantation development of the embryo and the period of implantation [13]. A commonality between these models is that the period of intervention extends beyond the periconceptional period to the postimplantation period and thus includes processes such as development of the uterine glands which provide nutrition (uterine histotroph) to the developing embryo and the process of placentation [14] Thus extension into the postimplantation period may not provide evidence of changes that are specific to manipulations during oocyte maturation and embryo development [15]. In contrast, the Adelaide model is restricted to the critical windows of oocyte maturation and preimplantation embryo development (60 days prior conception to 7 days after conception; term, 150 days) [16]. Similarly, the Southampton model also includes 15 days before until 15 days after conception (term, 147 days), which includes oocyte maturation and blastocyst formation [17].Therefore, isolating the impact of perturbations during oocyte maturation and the preimplantation period alone from those of the postimplantation and placentation periods [18,19,20].

## 3. Why Is Periconceptional Period a Critical Window of Development?

### 3.1. Oogenesis Occurs Early in Life

A women’s reproductive function is determined in fetal life because the specialized cells known as primordial germ cells (PGCs), that give rise to gametes, are present within the wall of the yolk sac at 4–6 weeks gestation [21,22,23]. PGCs migrate from the yolk sac to the genital ridge between 6 and 12 weeks gestation and differentiate into oogonia after becoming invested by somatic support cells [21]. By 12 weeks gestation, oogonia commence the first meiotic prophase and are called primary oocytes. Immediately after this, they become dormant in the early diplotene stage [23]. When the nuclei of these primary oocytes enlarge and become watery, the primary oocytes are known as germinal vesicles. The primary oocyte, surrounded by a single layered, squamous capsule of epithelial follicles is the primordial follicle [24]. At ~5 months gestation, the pool of these follicles reaches its peak of ~7 million, however, most of these follicles degenerate and the maximum number of oocytes a female will have in her lifetime is set at birth, ranging from 700,000 to 2 million [25,26], with only 400,000 follicles remaining at puberty [23,25]. Any changes in the maternal nutritional environment such as under- or overnutrition can affect oogenesis and other reproductive functions and hence can have an impact on the granddaughter’s reproductive health [27,28,29,30]. Studies have also shown that nutritional manipulations during pregnancy can have transgenerational effects, since they not only alter cardiovascular outcomes in the offspring but can also result in cardiovascular dysfunction and alter glucose and insulin control in the next generation [31,32,33,34]. Thus, in addition to understanding the impact of ARTs on the cardiovascular health of the offspring, it will be of interest to understand the impact on the grandchildren.

During and after puberty, hormonal secretions from the hypothalamus, anterior pituitary gland and ovary regulate the menstrual cycle, which averages 28 days in humans, 17 days in sheep and 4–5 days in rodents [35,36,37,38]. A small group of primordial follicles are converted into primary follicles with a decrease in inhibitory signals and/or an increase in stimulatory factors which remain largely unknown [39,40,41]. The granulosa cells undergo a squamous to cuboidal epithelial morphology from primordial to primary, and then several layers of granulosa cells begin to develop to form secondary follicle and the theca cells emerge in the transition from primary to secondary follicle [23,40]. A thick glycoprotein layer surrounds the surface of the oocyte known as zona pellucida [42]. Some of these follicles degenerate, but a few enlarge in response to rising levels of follicle stimulating hormone (FSH) and develop a fluid filled cavity known as the antrum from fluid generated by granulosa cell secretions and by plasma transudate (antral follicle) [43,44]. Eventually, one of the antral follicles becomes dominant (mature Graafian follicle), while the others degenerate [41]. At ~13–14 days of the menstrual cycle in humans, the primary oocyte resumes meiosis under the influence of the ovulatory surge of luteinizing hormone (LH) caused by positive feedback of estrogen, produced by the dominant follicle, on the pituitary gland and hypothalamus [23]. At the end of the first meiotic division, a small polar body containing a set of chromosomes is released into the perivitelline space [24]. Then the oocyte progresses to the second meiotic division, where it is again arrested in metaphase ~3 h before ovulation. Follicular rupture and ovulation occurs ~38 h after the beginning of the ovulatory surge [23]. The oocyte then moves into the ampulla of the oviduct and remains viable for fertilization for 24 h. Fusion of the sperm enables the oocyte to resume meiosis [45]. At the end of the second meiosis, the oocyte divides into two unequal cells: a polar body and the female pronucleus. The sperm loses its nuclear envelope, undergoes chromatin decondensation and replacement of the sperm specific protamine by histones. The DNA from the sperm binds to the histones in the oocyte and is surrounded by a new nuclear envelope of maternal origin, which forms the male pronucleus. The fusion of the pronuclei of sperm and oocyte results in a zygote [46]. Perturbations during oocyte maturation, such as those that may occur during parts of ART (ovarian hyperstimulation, *in vitro* maturation and *in vitro* fertilization (IVF)) have been shown to reduce the quality of oocytes and embryo viability as well as alter energy metabolism of the oocytes [47,48]. This has been shown to result in delayed embryonic development, increased abnormal blastocyst formation, fetal growth retardation, increased fetal loss, congenital malformations, imprinting disorders, and a range of postnatal growth and development disorders such as poor cognitive development, increased risk for neurological problems, cardiovascular diseases and respiratory tract infections [47,49,50,51,52,53,54]. In addition, superovulation can also perturb proper placental and fetal development by altering trophoblast differentiation and distribution of cell types in the placenta [55].

### 3.2. Key Events in Embryonic Development during the Periconceptional Period in Different Species

Studies in humans and animals have shown that nutritional manipulations during blastocyst formation, such as culturing the embryo in media, have been associated with cleavage anomalies, improper embryo development, an altered placental transcriptome, fetal and birth defects, increased blood pressure, vascular dysfunctions, poor neuromotor development, behavioural disorders and imprinting disorders [56,57,58,59,60,61,62,63]. In addition, the maturation of endometrium in human also takes place before implantation and studies have shown that ovarian stimulation could alter endometrial receptivity and impair implantation rate [64,65]. There is variation between species in the timing of ovulation after the beginning of estrous, completion of the second cleavage, hatching from the zona pellucida and implantation [66]. The steps from fertilization to implantation of the embryo include several major processes such as decondensation of parental genomes and cleavage to blastomere formation which includes an equal first cleavage (2 cell), and subsequent asynchronous division resulting in 4, 8, 16, 32 cell stages; (Table 1) [23,46]. With the development of a fluid filled cavity by the process of compaction, a blastocyst is formed [23]. The inner cell mass of the blastocyst will give rise to the embryo, the yolk sac, amnion and allantois while the outer cell mass develops into the chorion. As the hydrostatic pressure of the fluid increases within the cavity of the blastocyst, it expands and the zona pellucida is digested by enzymes which allow hatching of the embryo. This is followed by implantation of the embryo in the uterine wall (Table 1).

#### 3.2.1. Humans

Ovulation occurs in the middle of the menstrual cycle *i.e.*, around the 14th day of the cycle [67]. After fertilization, the formation of 2 cell (first cleavage), 4 cell, 8 cell and 16 cell (morula) stages take place at 24, 40, 50, 72 h, respectively. By the 5th day, the blastocyst is formed, followed by zona hatching at 5–7 days and implantation at 7–9 days (Table 1).

**Table 1:** Timing of important events in the periconceptional period in relation to ovulation [66,68,69].

Timing of events during the periconceptional period	Human	Mouse	Rat	Sheep
Time between ovulations	28 days	4–5 days	4–5 days	17 days
Time to 2 cell stage (first cleavage)	24 h	21–23	20.6 h	24–26 h
Time to 4 cell stage	40 h	38–50	72 h	30–36 h
Time to 8 cell stage	50 h	50–60	78 h	42–45 h
Time to 16–32 cells (morula stage)	72 h	60–70	84 h	63–86 h
Formation of blastocyst	5 days	3–4 days	4–5 days	5–6 days
Zona hatching	5–7 days	3.5	6 days	8 days
Implantation	7–9 days	4–5 days	6–7 days	16 days
Zygotic gene activation	40–50 h	24 h	24 h	30–45 h

#### 3.2.2. Rats

Ovulation in rats and mice occurs after the first two stages (proestrus and estrus) of estrous cycle, which is ~10 h after the start of estrus [70,71]. The embryo divides to form the 4 cell stage (second cleavage) by 38–50 h in mice and 72 h in rats, which is at a later time point than in humans. The blastocyst is formed at around 3–4 days in mice and 4–5 days in rats followed by hatching at day 3–3.5 in mice and at day 6 in rats. Implantation occurs at days 4–5 in mice and 6–7 in rats [72,73] (Table 1).

#### 3.2.3. Sheep

Ovulation occurs 20–30 h after the beginning of estrus [35]. After the fertilization, the first cleavage division occurs 24 h after ovulation (Table 2), followed by the 4, 8 and 16 cell stages at 30–36 h, 42–45 h and 63–86 h, respectively. The formation of the blastocyst occurs between 5 and 6 days after ovulation in sheep [12,74]. Hatching takes place at day 8, so that elongation can occur prior to implantation at day 16 [12].

### 3.3. Zygotic Gene Activation Occurs during Early Embryogenesis

One of the major events after fertilization is the transition of control of the developmental program of the zygote/embryo from maternally derived transcripts and proteins accumulated in the oocyte during the process of oogenesis to embryonic transcripts and proteins [75]. This transition is known as zygotic gene activation or embryonic gene activation [76]. Zygotic gene activation is associated with three main functions: (i) to degrade maternally inherited transcripts; (ii) to replace the oocyte specific transcripts that are common to both the oocyte and early embryo with zygotic transcripts; and (iii) to promote the generation of novel embryo specific transcripts by reprogramming the pattern of gene expression [68]. In humans and sheep the maternal-zygotic transition occurs between 4–8 cell stage and 8–16 cell stage respectively and is associated with the developmental loss of totipotency and in mice and rats, this occurs by the 2 cell stage (Table 1) [77]. The initiation of zygotic transcription also coincides with demethylation during embryogenesis, which is an important event in epigenetic programming that can affect chromatin structure and gene expression [78]. Nutritional manipulations such as culturing embryos in various media have been shown to delay the transcription of important growth factors such as platelet-activating factor-receptor in mouse preimplantation embryos, which can affect the viability of the embryo [79]. Studies have shown that IVF has differential effects on growth factors by either delaying the first onset of expression of some of growth factors after the activation of zygotic genome or by decreasing the expression, which can hamper proper embryogenesis in mice [80].

**Table 2:** Assisted Reproductive Technologies (ARTs) and manipulations during oocyte/embryo development [81,82].

ART Treatment	Infertility Treated	Procedures Involved and Manipulation of Oocyte/Embryo Development
IVF	Blocked Fallopian tubes, endometriosis, unexplained infertility, ovarian failure, ovulatory disorders and male infertility	*Controlled ovarian hyperstimulation*—Oocyte and follicular development
		*Oocyte retrieval and transfer*—Oocyte and follicular development
		*Sperm retrieval and preparation*—No direct effect on embryo
		*In vitro fertilization*—zygote
		*In vitro embryo culture*—zygote, cleavage, morula, blastocyst
		*Embryo transfer*—blastocyst
GIFT	Sperm dysfunction, endometriosis or unknown fertility	*Controlled ovarian hyperstimulation*—Oocyte and follicular development
		*Oocyte retrieval*—Oocyte and follicular development
		*Sperm retrieval and preparation*—No direct effect on embryo
ZIFT	Severe male infertility, immunologic infertility or unexplained infertility	*Controlled ovarian hyperstimulation*—Oocyte and follicular development
		*Oocyte retrieval*—Oocyte and follicular development
		*Sperm retrieval and preparation*—No direct effect on embryo
		*In vitro fertilization*—zygote
		*In vitro embryo culture*—zygote, cleavage
AI and IUI	Male infertility	No direct effect on oocyte development
ICSI	Male infertility	*Controlled ovarian hyperstimulation*—Oocyte and follicular development
		*Oocyte retrieval*—Oocyte and follicular development
		*Sperm retrieval and preparation—*No direct effect on embryo
		*In vitro fertilization*—Zygote
		*In vitro embryo culture*—Zygote, cleavage, morula, blastocyst
		*Embryo transfer*—Blastocyst

AI, artificial insemination; GIFT, gamete intraFallopian transfer; ICSI, intracytoplasmic sperm injection; IVF, *in vitro* fertilization; IUI, intrauterine insemination. ZIFT, zygote intraFallopian transfer.

### 3.4. Epigenetics Reprogramming Is an Important Event in Both Gametogenesis and Embryogenesis

**Figure 2:**
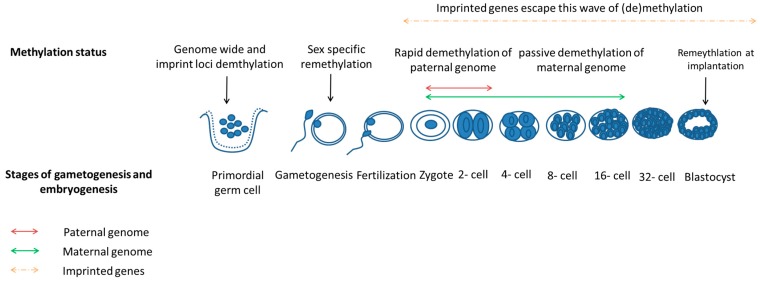
Epigenetic reprogramming during gametogenesis and embryogenesis. Adapted from [87,94].

Epigenetics is defined as all meiotically and mitotically heritable changes in gene expression that occur without changes in the DNA sequence [83,84]. One of the main mechanisms involved is DNA methylation (other mechanisms involved are histone modifications such as acetylation, phosphorylation, methylation, ubiquitination and sumoylation) [85,86]. During mammalian development, gametogenesis and embryogenesis are the two critical periods where epigenetic reprogramming occurs (Figure 2) [87]. This begins with demethylation when PGCs migrate along the genital ridge followed by sex-specific pattern of remethylation before fertilization [88,89]. There is a second wave of whole genomic demethylation in the male pronucleus within hours of fertilization whereas the female pronucleus undergoes complete demethylation after several cleavage divisions [90]. During implantation, genome-wide methylation takes place in a lineage-specific pattern [89]. However, there are certain genes, known as imprinted genes, that undergo erasure of methylation marks during PGC development and the methylation marks are re-established during gametogenesis but escape the second wave of demethylation that occurs after fertilization and thus maintain their methylation of CpG islands that was established during gametogenesis [91,92]. Imprinted genes are expressed differentially depending on their inheritance from maternal or paternal origin [93]. During development, one of the alleles of a particular gene is expressed only in the zygote and the other allele is silenced, and this process is known as genomic imprinting [87].

This epigenetic reprogramming is essential for proper development because it controls expression of early embryonic genes, cell cleavage and cell determination [95]. Imprinted genes, in particular, play an important role in embryonic and fetal development as well as placental function [96,97,98]. Any defects during epigenetic reprogramming, including the imprinting process, may affect genotype and phenotype and are linked to embryonic abnormalities and diseases in later life [99]. As such, the periconceptional period, which involves the crucial process of epigenetic remodelling, makes the early oocyte or the preimplantation embryo vulnerable to perturbations that can have immediate and long-term consequences on the health of the offspring. For example, there is compelling evidence for a link between ARTs, manipulations in the periconceptional period, and epigenetic disorders (imprinting defects) [100]. A recent study has also shown that maternal nutritional status during the periconceptional period was associated with persistent epigenetic changes at human metastable epialleles [101].

This section has provided evidence that the periconceptional period encompasses many important events that determine health of the embryo and that any manipulation during this period, such as ART, can impact the processes of oocyte maturation, embryogenesis, fetal and postnatal development. It is important to understand that different types of ARTs are being used and thus that each type of ART involves different manipulations to the periconceptional period.

## 4. What Are the Different Procedures Involved in ARTs and How Do These Impact the Periconceptional Environment?

### 4.1. History, Procedures and Current Status of ARTs

ARTs are manipulations during the periconceptional period in which the oocyte, sperm and/or zygote develop that apply laboratory or clinical techniques to gametes (sperm and ova) and/or embryos to establish pregnancy [102,103,104]. The history of ARTs dates back to 1890 when the first successful embryo transfer occurred in rabbits [105]. A live calf was successfully produced from embryo transfer 60 years later [106]. In 1973, two of the earliest human pregnancies occurred at the Queen Victoria Hospital in Melbourne but they lasted for less than a week [107]. These were chemical pregnancies identified by increasing plasma human chorionic gonadotropin (hCG) concentrations and provided the evidence that embryos produced from IVF could develop to the blastocyst stage and initiate implantation. The birth of the world’s first test-tube baby, Louise Brown, occurred on 25 July 1978 at Oldham hospital in England, UK [108,109]. This was a landmark in the area of reproductive medicine and paved the way for scientific developments to improve implantation and pregnancy rates.

The use of ARTs has increased rapidly around the globe in recent years. In 2011, 588,629 treatment cycles were performed at clinics in 33 European countries, 151,923 cycles in the US and 66,347 cycles in Australia and New Zealand [110]. According to the European Society of Human Reproduction and Embryology, an estimated 5 million children have been conceived as result of ARTs [110]. The extensive usage of ARTs is attributed to increasing infertility rates in the population [111,112]. One of the reasons could be that women are considering pregnancy later in life. The average age of a first pregnancy has increased from 26.9 years in 1983 to 30.1 years in 2012 in Australia [113,114]. In 1991, around 23% of new mothers were aged over 30 years but this has risen to 37% in 2001 and 43% in 2011 [115]. Similar trends were observed in the USA and Canada where the average age of women having their first child increased from 21.4 years in 1970 to 25.8 years in 2012 and from 23.5 in mid 1960s to 28.1 in 2008, respectively [116,117]. There is evidence to suggest that fertility as well as fecundity declines with increasing age for both men and women [112,118,119,120]. Delayed child-bearing also results in increased time to conception and complications during pregnancy [121]. With recent advancements in both ARTs and clinical practice, coupled with the widespread availability of ART and improved success rates, there has been a rise in the number of couples that are experiencing problems with conception seeking treatment [122].

The improvements in ARTs have also benefited the livestock industry, which have resulted in great advancements in livestock production [123,124]. The introduction of reproductive technologies has increased production as well as reproduction of livestock animals with desired genetic characteristics [124,125]. They have also helped in overcoming reproductive problems, such as reducing generation intervals and preventing the spread of vertically transmitted diseases [126,127]. Livestock industries as a whole have scaled to new heights in terms of economic gains with the help of these reproductive technologies [125,128].

The most widely used ART is IVF because it has the highest success rate per cycle, irrespective of the cause of the infertility [81]. It was developed to treat infertility associated with blocked Fallopian tubes and to treat other disorders related to infertility such as endometriosis, unexplained infertility, ovarian failure, ovulatory disorders and male infertility [129,130]. Gamete intraFallopian transfer (GIFT) and zygote intraFallopian transfer (ZIFT) were developed as alternative treatments to IVF in specific circumstances [82,131]. GIFT involves introduction of both egg and sperm in the Fallopian tube where fertilization and subsequent embryo development occurs [132,133,134,135]. GIFT treatment can be used as a treatment for sperm dysfunction, endometriosis or unknown infertility [136,137], but requires that at least one Fallopian tube is open. ZIFT involves removal of eggs followed by IVF with subsequent transfer of the embryo at the 2 cell stage into the Fallopian tube [138]. It can be used in cases of severe male infertility, immunologic infertility or unexplained infertility [138,139,140,141]. Other ARTs include artificial insemination (AI), intrauterine insemination (IUI) and intracytoplasmic sperm injection (ICSI), which are generally used in cases of male infertility and can be coupled with other ARTs, such as IVF, depending on the severity of the infertility (Table 2) [142,143].

### 4.2. Periconceptional Manipulations Associated with ARTs

A typical IVF treatment involves administration of FSH for controlled ovarian hyperstimulation over several days to induce the development of multiple follicles and the maturation of oocytes is induced by administration of hCG. This is followed by aspiration of mature oocytes from the ovarian follicles and insemination with a prepared sperm sample *in vitro*. The zygote that forms after fertilization of the oocyte and sperm is cultured in a nutritional medium for either 2–3 days to form a cleavage stage embryo or 5–6 days to form a blastocyst, which is then transferred to the uterine cavity for implantation (Table 2) [144,145]. In the past, more than one embryo was transferred to increase the chance of pregnancy and this has been associated with multiple pregnancies [146]. Twin pregnancies are at an increased risk of premature birth and its associated complications [147,148]. Therefore, transfer of a single embryo has been advocated to reduce the risk of multiple pregnancies [149]. With the transfer of a single embryo, a desirable pregnancy rate with fewer multiple pregnancies has been achieved [150]. However, even with the transfer of a single embryo, there are reports of increased twinning [151].

### 4.3. In Vitro Embryo Culture: A Periconceptional Manipulation of the Nutritional Environment of the Gametes and/or Zygote Associated with ARTs

An important component of many ARTs is the nutritional medium used for culturing the embryo [152,153]. The nutritional medium contains essential components required for optimal growth and to maintain the viability of the embryo at various stages of development [154]. The culture media from different clinics varies in composition but can be broadly classified into simple and complex media [155]. Simple culture medium includes Tryode’s, Earle’s T6, M16 and CZB, which contain balanced salt solutions and energy sources such as pyruvate, lactate and glucose. These are generally supplemented with serum from the patient or fetal cord serum because the media are known to lack of components that are essential for the growth and development of the embryo [156].

Efforts have been made to design media to match the dynamic *in vivo* environment and have led to the development of human tubal fluid (HTF) media [157]. HTF mimics the physiological environment of the human Fallopian tube and is based on the chemical composition of human tubal fluid [153,157]. The complex tissue culture medium contains amino acids, vitamins, nucleic acid precursors and metal ions and was originally designed to support somatic cells in culture, e.g., Ham’s F-10, MEM and TCM-199 [155]. In recent years, media were designed with particular focus on each of the developmental stages of the embryo, broadly divided into pre-compaction and post-compaction stages or the cleavage and blastocyst stages [156].

The inclusion of human serum, which acts as a pH buffer and chelator for embryonic toxins such as “transitional and heavy metals” in addition to providing growth factors and nutrients such as amino acids and vitamins in the media, has been long debated [158,159,160]. The use of human serum in different culture media during the first 2 days of embryonic development in sheep leads to retarded growth but its inclusion during later stages leads to more blastocysts formations [161]. However, there are also reports of altered ultrastructure of the mitochondria and energy metabolism, epigenetic disorders including errors in imprinting, abnormal growth and gross abnormalities in several organ systems as a result of the addition of serum in the media during the early stages of embryonic development [162,163,164,165]. Recently, serum free culture media have been developed and proven to yield better results with increased blastocyst formation, higher implantation rates and reduced risk of contamination of the media by unknown components present in the serum [166,167].

We have described the complex maturation and developmental events of the oocyte and embryo that occur in the periconceptional period. These events must occur appropriately to allow gastrulation and the appropriate allocation of cells to each of the developing organ systems. We have also described the nutritional manipulations that the gametes and zygote are exposed to during the processes of ARTs (Table 2). Together, this information leads to questions about whether there is evidence that the periconceptional environment influences cardiovascular health as a fetus and into adulthood.

## 5. What Is the Evidence that the Periconceptional Environment Influences Cardiovascular Health in Fetal Life and Adulthood?

Cardiovascular disease is the leading cause of death worldwide causing an estimated 17.5 million deaths worldwide in 2012 and it is estimated to reach 23.3 million by 2030 [168,169]. Cardiovascular disease in adults is generally attributed to an unhealthy lifestyle during adulthood with most focus on poor diet, a sedentary lifestyle and smoking [170,171]. However, a growing body of evidence suggests that cardiovascular and metabolic disorders in adulthood may derive their origins from insults during prenatal life, including the periconceptional period [20,172]. Barker and colleagues coined the Fetal Origins of Health and Disease hypothesis, which stated that changes in the development of a permanent somatic structure or the “setting” of a physiological system by an early stimulus or insult during a critical period of development during which any manipulation of either environmental or nutritional factors can have long-lasting consequences on the physiology of the embryo and fetus [173,174].

Studies have shown that normal development of the mammalian cardiovascular system during the embryonic period as well as the transition from proliferative to hypertrophic cardiomyocytes growth during late gestation is dependent on the timely and accurate activation of many genes and signalling pathways [59,175]. Some of these signalling pathways are under epigenetic regulation such as DNA methylation and histone modifications [176,177]. Any abnormalities in the epigenetic control of these processes may result in cardiovascular malformation and susceptibility to disease in adult life [178,179].

The heart is comprised of cardiomyocytes and non-myocytes including fibroblasts, endothelial cells, mast cells, vascular smooth muscle cells and the surrounding extracellular matrix [180,181]. During the first two thirds of gestation, heart growth is predominantly due to proliferation of mononucleated cardiomyocytes [182]. In humans and sheep, cardiomyocyte maturation, characterised by quiescence (absence of cardiomyocyte cytokinesis), occurs in the last third of gestation and subsequently heart growth in late gestation and postnatal life is primarily through cardiomyocyte hypertrophy [183,184]. In sheep, cardiomyocyte quiescence is easily identified due to almost all mononucleated cardiomyocytes becoming binucleated and subsequently terminally differentiated by birth [175,185,186,187]. In humans, the majority of cardiomyocytes remain mononucleated, however, it is widely accepted that only a tiny proportion of mononucleated cardiomyocytes undergo cytokinesis/proliferation after birth [188,189]. Recent studies have also provided evidence that cardiomyocytes continue to proliferate until almost 20 years of age [190]. In rodents, all cardiomyocytes are mononucleated at birth and retain the ability to proliferate and regenerate cardiac tissue after damage within the first week of life [191,192]. Like sheep, rodent cardiomyocytes become binucleated and terminally differentiated, however, unlike sheep and humans, this occurs rapidly between 4 and 12 days after birth [192,193,194]. Due to the limited potential for cardiomyocyte proliferation in postnatal life, the heart has a limited capacity to replace cardiomyocytes that are lost due to disease and aging. Subsequently, the number and the epigenetic profile of cardiomyocytes an individual is born with will have lifelong implications for cardiac health.

### Periconceptional Manipulations and Cardiovascular Disorders: Insights from Human and Animal Studies

A series of epidemiological, clinical and experimental studies have shown that nutritional manipulations during the periconceptional period, may have adverse effects on cardiovascular health (Table 3) [59]. The most profound evidence was from the Dutch Winter Famine of 1944/45, which demonstrated that offspring exposed to malnutrition as an embryo or fetus during early gestation had elevated blood pressure in response to physiological stressors and 8.8% of those exposed during early gestation also had an increased risk of coronary heart disease compared to 0.9% and 2.5% of offspring whom were exposed to the famine in mid or late gestation respectively [195,196]. In another cohort periconceptional maternal tobacco smoking was associated with increased risk of congenital heart defects such as septal defects [197,198]. Likewise, alcohol consumption during the periconceptional period elevated conotruncal heart defects in offspring and that risk was associated with the frequency and the number of drinks consumed [199]. In a study examining the effects of the interaction of genetic factors and periconceptional nutritional manipulations on congenital heart defects, it was found that a low maternal dietary nicotinamide intake and usage of medicines such as antibiotics, anticonvulsants, anti-inflammatory, hormones and antimycotics during the periconceptional period independently increased the risk of congenital heart defects in the offspring by 2 fold [200].

**Table 3:** Maternal undernutrition during the periconceptional period alters cardiovascular development.

Species	Periconceptional Manipulation	Time	Blood Pressure	Baroreflex Sensitivity	Congenital Heart Defects	Risks for Heart Diseases	Heart Weight	Vaso-Constriction	Vasodilation
Human	Undernutrition	Early gestation	↑ [196]	n/a	n/a	↑ [195]	n/a	n/a	n/a
	Alcohol consumption	−30–+90 days	n/a	n/a	↑ [199]	n/a	n/a	n/a	n/a
	Low maternal dietary nicotinamide and exposure to a range of medicines	−30–+60 days	n/a	n/a	↑ [200]	n/a	n/a	n/a	n/a
Rodent	Protein restriction	0–4.25 days	↑ [201]	n/a	n/a	n/a	n/a	n/a	n/a
	Low protein diet	−3.5–0 days	↑ [202]	n/a	n/a	n/a	n/a	n/a	↓
		0–3.5 days	↑ [203]	n/a	n/a	n/a	↓ [203]	n/a	n/a
Sheep	Maternal undernutrition	−60–7 days	↑ [204]	n/a	n/a	n/a	n/a	n/a	n/a
		1–30days	↔ [205]	↓[205]	n/a	n/a	n/a	n/a	n/a
		0–95 days	↑ [206]	↓ [206]	n/a	n/a	n/a	n/a	n/a
		−30–0 days	n/a	n/a	n/a	n/a	n/a	n/a	↓ [17]
		−15–15 days	n/a	n/a	n/a	n/a	n/a	↑ [17]	↓ [17]
		1–31days	↑ [207]	n/a	n/a	n/a	↑ [207]	↑ [207]	n/a
		−61–30 days	n/a	n/a	n/a	n/a	↑ [11]	n/a	n/a
		−61–0 days	n/a	n/a	n/a	n/a	↓ [208]	n/a	n/a
		−61–30 days	n/a	n/a	n/a	n/a	↓ [208]	n/a	n/a
		−2–30 days	n/a	n/a	n/a	n/a	↓ [208]	n/a	n/a

↑ = increase, ↓ = decrease, ↔ = no difference, n/a = not applicable (because not included in the reported study results).

Animal models have been useful in identifying the underlying mechanisms that are responsible for the association between nutritional manipulation in the periconceptional period and cardiovascular health in adult life (Table 3). Maternal protein restriction during the preimplantation period in rats resulted in a reduced number of cells in the blastocyst stage, a reduction in birth weight, accelerated postnatal growth and elevated systolic blood pressure (SPB) in postnatal life [201]. Maternal protein restriction, specifically during oocyte maturation, led to hypertension in the adult offspring [202]. Similarly maternal protein restriction from 0 to 3.25 days after mating reduced heart weight and increased SPB in offspring in postnatal life [203]. In addition, the heart to body weight ratio in females was negatively correlated with SBP measured at 9, 15, and 21 weeks [203].

In sheep, maternal undernutrition during the periconceptional period extending from 60 days prior to conception until 7 days after fertilization resulted in an increase in arterial blood pressure and rate pressure product in twins, but not singleton fetuses in late gestation (term, 150 days) [204]. There was altered baroreflex sensitivity in response to angiotensin II infusion at 1 year of age as a result of global energy restriction from the day of conception until 30 days of gestation [205]. Maternal undernutrition extending from 0 to 95 days gestation resulted in an increase in prefeeding basal blood pressure and blunted baroreflex in response to norepinephrine infusion at 3 years of age [206]. In another study undernutrition during the 30 days before conception resulted in diminished endothelium-dependent and independent vasodilatation in third order femoral arteries [17]. An undernutrition regime from 15 days prior to until 15 days after conception resulted in greater vasoconstrictor responses in both left anterior descending coronary and left internal thoracic arteries [17]. There was attenuated endothelium-dependent and independent vasodilatation in third order femoral arteries as well as reduced endothelium-independent vasodilation in both the left anterior descending coronary and renal arteries [17]. There was an increase in blood pressure in response to frusemide, a loop diuretic used to activate renin angiotensin system, in 1.5 year old lambs of ewes that were undernourished between 1 and 31 days of gestation. At 2.5 years, these lambs had increased interventricular septal wall thickness, mean left ventricular wall thickness and increased constriction to acetylcholine in isolated coronary arteries [207]. Furthermore, offspring born to ewes that were undernourished from −61 days before until 30 days after conception had increased fetal heart weight relative to body weight in late gestation [11]. In contrast, relative heart weight was reduced in the offspring of ewes that were undernourished from 60 days prior to conception, 60 days before and 30 days after conception and 2 days before and after conception [208].

The above studies provide evidence that nutritional manipulation in the periconceptional period has adverse effects on the cardiovascular system, which might predispose an individual to an increased risk of cardiovascular disease.

## 6. What Is the Evidence that ARTs Influence Cardiovascular Health Before and After Birth?

Studies have demonstrated that not only insults during the periconceptional period *in vivo* (Table 3), but also those that occur *in vitro* during this critical period of development can have detrimental effects on cardiovascular development [13,59,204,209,210]. This highlights the importance of understanding the long-term effects of ARTs on cardiovascular development. There are reports of altered fetal and postnatal growth and development as a result of *in vitro* nutritional manipulations during the periconceptional period in humans, rodents, cows and sheep [211,212,213]. These reports have raised serious questions about the safety of ARTs for both the immediate and long-term health of individuals conceived through ARTs. Among the long-term health outcomes, cardiovascular health remains the most important concerns due to the sensitivity of heart development to perturbations during the periconceptional period [20,59,214,215].

A recent study reported the incidence of cardiac malformations using different search terms related to cardiac malformations and/or ARTs in the Medline database for the years 1999 to 2012. The search returned data on 32,000 births (21,000 naturally conceived; 11,000 conceived through ART) [216]. Cardiac malformations occurred in 1.8% (198 cases) of births conceived through ART compared to 0.4% (88 cases) of births in the general population [216]. Comparing the incidence of specific forms of cardiovascular disorders from a total of 11,000 pregnancies in ART children and 21,000 pregnancies in naturally conceived (NC) children respectively, there were 58 cases (0.53%) of atrial septal defect in ART *vs.* 26 cases (0.12%) in NC, 37 cases (0.34%) of changes in the interventricular septum in ART *vs.* 18 cases (0.09%) in NC, 20 cases (0.18%) of coarctation of the aorta *vs.* 13 cases (0.06%) in NC, 18 cases (0.16%) of aortic stenosis in ART *vs.* 11 cases (0.05%) in NC, 11 cases (0.10%) of tetralogy of Fallot in ART *vs.* 5 cases (0.02%) in NC, 6 cases (0.05%) of stenosis of the pulmonary trunk in ART *vs.* 5 cases (0.02%) in NC and 48 cases (0.44%) of other non-specified cardiac defects in ART and vs 10 cases (0.05%) in NC. [216]. These data suggest that compared to natural conception, there is an increased risk of cardiovascular malformations in children conceived through ART.

### 6.1. Congenital Heart Defects in Human Population

Many studies have reported the incidence of congenital heart defects in children conceived through ARTs (Table 4). In the first report of a relationship between ART and congenital heart defects, Lancaster *et al.* found that there were 4 cases of transposition of the great arteries in the offspring conceived through IVF [217]. Several studies have also confirmed these findings. For example, a significant increase in overall congenital heart defects in children conceived through IVF compared to children with unassisted conception was reported in different population based studies [218,219,220,221]. The increase in cardiovascular defects also remained significant when the analysis was performed in only singletons [218,219]. Another population based study in Finland compared the risk of congenital defects in IVF and other ART categories with the control population based on infant sex and multi-fetal pregnancies and found an increase in congenital heart defects in the female offspring of singleton pregnancies in other ART categories group [222].

A case-control study using the California Linked Birth Cohort Dataset found an increased risk of major cardiac malformations in 4.8% of births from ART (IVF with or without ICSI, GIFT) compared to 3.0% in the matched control population [50]. After adjusting for maternal and infant factors such as maternal age, parity, race, multiple births, infant sex and year of birth, the rate of congenital heart defects was significantly higher in the infants born after ARTs [50]. When the heart defects in singleton and multi-fetal ART pregnancies were compared, the multi-fetal pregnancies had an increased risk of congenital cardiac defects than the singleton pregnancies [50]. An increased rate of congenital malformations in infants conceived by ART was also reported in a retrospective cohort study in Ottawa [223]. In a cross-sectional descriptive study in Iran, 8 cases of IVF pregnancies with congenital heart defects were reported in a population of 400 ART children [224].

In addition to evidence that ARTs increase the risk of cardiac defects, studies have identified specific congenital cardiac defects associated with ARTs (Table 4). The prevalence of congenital heart malformations such as atrial septal defects and ventricular septal defects was four-fold higher in children born after IVF than the matched controls in a Finnish population based cohort study [225] In a cross-sectional study in Iran, children conceived through ARTs had increased risk of ventricular and septal defects [226]. A population based study analysed specific cardiovascular defects in the Danish population study and reported a significant increase in the prevalence of a single umbilical artery in IVF children when compared to controls [221]. In another population based, multicenter case controlled study of birth defects in the United States, the singleton births resulting from IVF and ICSI were at increased risk of septal heart defects, including ventricular septal defect, atrial septal defect and other non-specified defects compared to unassisted conception [227]. Using data from a Swedish database for the period 1982–2001, it was found that in addition to an overall risk of congenital heart diseases, the association between ARTs and congenital heart defects became more marked when the analysis was restricted to major cardiac defects such as common arterial trunk, double outlet right or left ventricle, D- and L-transposition of great vessels, double inlet left ventricle, endocardial cushion defect, tetralogy of Fallot, tricuspid atresia or stenosis, Ebstein’s anomaly, hypoplastic left heart syndrome, aortic valve atresia, specified anomalies of the great veins or to septal defects [228]. In another study, which used the Swedish database for the period 2001–2007 found an increased risk of cardiovascular malformations, which was similar to the data from the same database for the period (1982–2001) [229]. The study also reported an increased risk of septal and ventricular defects but the risk was lower during the period 2001–2007 compared to the previous time period [229].

Based on a case-control study using data from Paris Registry of Congenital Malformations, it was found that cases of congenital heart diseases were more likely to be born as a result of ARTs when compared to controls [230]. The study investigated three major categories of congenital heart defects (all congenital heart diseases combined, congenital heart diseases without chromosomal abnormalities and congenital heart diseases without chromosomal abnormalities (excluding isolated ventricular septal defects)) and it was found that there was also a 40% increase in the overall risk of congenital heart disease without chromosomal abnormalities in infants conceived through ARTs after adjusting for maternal age, socioeconomic factors and year of birth [230]. Specific congenital malformations such as malformations of the outflow tracts and ventriculoarterial connections, cardiac neural crest defects and double outlet right ventricle were significantly associated with ARTs [230]. The study also reported that IVF and ICSI were associated with an increased risk of congenital heart disease, but not ovulation induction alone [230]. Another case–control study using data from the population-based Paris Registry of Congenital Malformations, and a prospective cohort study of congenital heart disease in children (EPICARD) investigated the association between ARTs and four specific forms of congenital heart diseases (hypoplastic left heart syndrome, transposition of great arteries, tetralogy of Fallot, and coarctation of the aorta) [231]. The study found that ARTs (IVF, ICSI and ovulation induction alone) were associated with a 2.4-fold increased risk of tetralogy of Fallot, after adjusting for maternal age, occupation, geographic origin, paternal age and year of birth [231]. There was no significant association between ART and the other 3 congenital malformations [231].

### 6.2. Evidence of Risk Factors for Long-Term Cardiovascular Outcomes in Humans

The increased risk of cardiovascular defects at birth provides a strong justification for follow-up studies to investigate the effect of the procedures involved in ARTs on the long-term consequence on cardiovascular health outcomes. What is not known is whether ARTs are associated with an increased risk for clinical cardiovascular endpoints due to the young age of the ART population in humans. However, emerging studies have provided evidence for an association of ARTs with increased risk factors for cardiovascular disease (Table 5).

Fetuses from ART pregnancies had altered cardiac morphometry and impaired systolic and diastolic function compared to controls [232]. When they were assessed during neonatal life, an increased diastolic blood pressure percentile, aortic and carotid intermedia thickness was reported in ART children compared to controls [232]. During infancy, children from ART pregnancies had increased right atrial size, right ventricular wall thickness, aortic wall thickness, SBP and heart rate as well as decreased shortening fraction, right sphericity index and systolic and diastolic function [232]. This evidence suggests that cardiovascular remodelling was present in fetal life and persisted into infancy in ART children and this may suggest an increased risk of cardiovascular disease in later life.

A study has also shown that the children conceived through IVF had increased SBP and DBP at a mean age of 12.3 years [233]. Furthermore, there was an association between early childhood weight gain and SBP during follow-up [234], which is an important risk factor for cardiovascular disease in later life [235,236]. Similarly, in a cross-sectional study in Athens, a higher SBP and DBP deviation score was reported in ARTs children compared to naturally conceived children in the age group 4–14 year [237].

Vascular functions were studied in ART conceived children at around 12 years of age and a ~25% reduction in flow-mediated dilation of the brachial artery evoked by reactive hyperemia was reported but no difference was observed with endothelium-independent vasodilation evoked by nitroglycerine [238]. This finding is suggestive of endothelial dysfunction in ART offspring, which is an important and early marker for the development of atherosclerosis in later life and has previously been shown to be present in healthy children whom were at increased risk of cardiovascular diseases [239,240,241,242]. The study also found a significant faster carotid femoral pulse wave velocity, which is widely used as a gold standard for measuring arterial stiffness as well as an increased carotid intima-media thickness, both of which are markers for development of atherosclerosis and independent risk factors for cardiovascular disease [238,243,244,245]. In addition, ART children had 30% higher pulmonary pressure at higher altitude [238]. The findings from this study provide evidence for both systemic and pulmonary vascular dysfunction in ART children, which could lead to adverse cardiovascular outcomes in later life [238]. Most importantly, it was reported that the vascular dysfunctions were not related to parental factors but more likely due to the ART procedure itself.

**Table 4:** Congenital heart defects that result from ART.

Study Type; Population; Year	Sample Size (*N*)	Congenital Heart Defects		Reference
Registry of IVF and GIFT pregnancies in Australia and New Zealand	IVF-1694	4-cases of transposition of great arteries (*p* = *0.0034*)*.*		[217]
Population based study; Children conceived by IVF or IUI at the University of Iowa; 1989–2002	IVF-1462 IUI-343 Controls-8422	- Increased cardiovascular defects among the infants conceived through IVF when compared with control children (*p* = 0.002) but no significant increase in cardiovascular defects in IUI infants. - Significant increase in cardiovascular defects was also reported when the analysis was done only in singletons (*p* = 0.003).		[218]
Population-wide cohort study; South Australian Perinatal Statistics Collection; January 1986–December 2002	ART-6163 Controls-302,811	- There was significant association between the use of ARTs and risks of multiple cardiovascular defects for singleton births *(1.8% vs. 1.2%; adjusted OR 1.36 (1.08–1.72))*. - (Adjusted for maternal age, parity, fetal sex, year of birth, maternal race or ethnic group, maternal country of birth, maternal conditions in pregnancy, maternal smoking during pregnancy, socioeconomic status, and maternal and paternal occupation)		[219]
Population based study; Reproductive Technology Register; 1993–1997	IVF-837 ICSI-301 Controls-4000	Increased prevalence of cardiovascular defects in children conceived with IVF, but not those conceived with ICSI compared to controls (*p* *<* 0.001).		[220]
Population based study; National professional Perinatal and Neonatal Registers; 1995–1996	IVF-4224 Controls-314,605	- Increased risk of overall cardiovascular malformations *(OR 1.56, 95% CI 1.10–2.2)*. - After analysing specific cardiovascular malformations, an increased risk of single umbilical artery was reported *(OR 1.93, 95% CI 1.11–3.35)*. (Adjusted for maternal age, parity and ethnicity)		[221]
Population based study; Medical Birth Register, Finland; 1996–1998	IVF-4559 Other ARTs-4467 Controls-27,078	- Increased risk of congenital heart defects in both IVF (*p* = 0.042) and ART categories (*p* = 0.021) compared to controls when expressed as prevalence of birth defects per 10,000 infants. - The singleton girls in other ART categories had an increased incidence of congenital heart defects when analysis was done for gender and multiplicity *(adjusted OR 1.52, 95% CI 1.01–2.28)*. (Adjusted for age)		
Case-control; California Patient Discharge Linked Birth Cohort Database Dataset; 2006 to 2007	ART-4795 Controls-46,025	- Born after ARTs *(adjusted OR 1.41, 95% CI 1.22–1.64)*. - The multi-fetal pregnancies were at increased risk of congenital cardiac defects compared to singleton *(adjusted OR 1.56, 95% CI 1.31–1.85)*. (Adjusted for maternal and infant factors such as maternal age, parity, race, multiple births, infant sex and year of birth)		[50]
Retrospective cohort study: Ottawa; Fertility Centre; 1996–2005	ART-1044 Controls-1910	- Higher rate of congenital heart defects in infants conceived by ART than controls *(adjusted OR 4.58, 95% CI 1.48–14.18).* (Adjusted for maternal age, plurality, year of delivery, catchment area, gestational weight gain, parity, maternal smoking, medical history, Rh. negative, pelvic surgery)		[223]
Cross-sectional descriptive study; Royan Institute, Tehran;	ART-400	8 cases (2%) of defects in cardiovascular system.		[224]
Population based cohort study; IVF Outpatient Clinic, University of Oulu and Infertility Clinic of the Family Federation of Finland and Oulu Controls- Finnish Medical Birth Register; 1990–1996	IVF-304 Controls-569	4 fold increase in atrial septal and ventricular septal defects *(OR 4.0, 95% CI 1.4–11.7).*		[225]
Historical cohort study; Child Health and Development Research Centre; January 2008–December 2010	ART-326 Controls-652	Increased risks of cardiovascular malformations in ART children *(1.07% vs. 1.53%)*.		[226]
Population based, multicenter case-control study, National Birth Defects Prevention Study; October 1997–December 2003	Cases-9584 (with birth defects) Controls-4792 (no birth defects)	- Increased risk of overall septal heart defects (ventricular septal defect, atrial septal defect and other non-specified defects) in singleton infants born after ART compared to unassisted conception *(adjusted OR 2.1, 95% CI 1.1–4.0).* - Increased risk of atrial septal defect secundum/ not otherwise specified defects *(adjusted OR 3.0, 95% CI 1.5–6.1)*, and ventricular septal defect plus atrial septal defect *(adjusted OR 2.8, 95% CI 1.2–7.0)* in singleton infants compared to unassisted conception. (Adjusted for maternal age, study center, parity, family income and prematurity)		[227]
Population based study; Swedish Medical Birth Register, Swedish Registry of Congenital Malformations, and Swedish Hospital Discharge Register; 1982–March 31, 2001	ART-16,280 Controls-2,039,943	- An overall increased risk of congenital heart defects in infants conceived through ART was observed compared to controls; *(adjusted OR 1.7, 95% CI 1.5–2.0)*. - A stronger association between ARTs and congenital heart defects when the analysis was restricted to major cardiac defects such as common arterial trunk, double outlet right or left ventricle, d- and l transposition of great vessels, double inlet left ventricle, endocardial cushion defect, tetralogy of Fallot, tricuspid atresia or stenosis, Ebstein’s anomaly, hypoplastic left heart syndrome, aortic valve atresia and specified anomalies of great veins *(adjusted OR 2.1, 95% CI 1.6–2.8)* or to septum defects in the absence of non-cardiovascular defects *(adjusted OR 2.6, 95% CI 2.2–3.1)*. (Adjusted for year of birth)		[228]
Population based study; Swedish Medical Birth Register, Swedish Registry of Congenital Malformations, and Swedish Hospital Discharge Register; 31 March 2001–1 January 2007	IVF-15,570 Controls-5,689,157	- An overall increased risk of cardiovascular defects in infants conceived through ART was observed compared to controls *(adjusted OR 1.30, 95% CI 1.13–1.49).* - Compared to a previous study using the same database from 1982–2001, it was reported that both the studies had increased risk of atrial septal defects and ventricular septal defects *(OR 3.16, 95% CI 2.71–3.67) vs.* *(OR 2.35, 95% CI 2.09–2.64)*. (Adjusted for year of birth, maternal age, parity, smoking, and body mass index)		[229]
Case control study; Paris Registry of Congenital Malformations; 1987–2006	Cases-5493 (with congenital heart defects) Malformed controls-3487 (malformations not previously associated with ART)		- Cases from congenital heart diseases were more likely to be conceived through ARTs than other malformed controls *(4.7% vs. 3.6%; p =* 0.008*).* - Infants conceived through ART have 40% increase in the overall risk of congenital heart disease without chromosomal abnormalities. - Significant associations between ARTs and specific congenital malformations such as malformations of the outflow tracts and ventriculoarterial connections *(adjusted OR 1.7, 95% CI 1.2–2.4)* as well as cardiac neural crest defects and double outlet right ventricle *(adjusted OR 1.7, 95% CI 1.1–2.7)*. (Adjusted for maternal age, geographic origin, occupation, and year of birth)	[230]
Case-control; Paris Registry of Congenital Malformations; 1987–2009. Prospective cohort study, congenital heart disease in children (EPICARD); 2005–2008	Case-1583 (with congenital heart defects) Malformed controls-4104 (malformations not previously associated with ART)		- ARTs were associated with a 2.4-fold increased risk of tetralogy of Fallot *(adjusted OR 2.4, 95% CI 1.5–3.7)*. - No significant association between ART and hypoplastic left heart syndrome, transposition of great arteries and coarctation of the aorta malformations. (Adjusted for maternal age, occupation, geographic origin, paternal age and year of birth)	[231]

OR, odds ratio; CI, confidence intervals.

**Table 5:** Cardiovascular risk factors in fetal and postnatal life.

Study Type; Population; Year	Sample Size (*N*); Age(s)	Cardiovascular Risk Factors	Reference
Prospective cohort study; Maternal-Fetal Medicine Unit, Spain.	ART-100 Controls-100 28–30 weeks gestation, 1 month, 6 months	- Fetuses (28–30 weeks gestation)—Increased left atrial area *(1.48 vs. 1.35; p* < 0.001*)*, right atrial area *(1.60 vs. 1.46; p* < 0.001*),* interventricular wall thickness *(2.7 vs. 2.4; p* < 0.001*)* and right wall thickness (3.2 *vs.* 2.8; *p* = 0.038), decreased left *(*1.71 *vs*. 1.77; *p* = 0.003*)* and right *(*1.37 *vs.* 1.58; *p* < 0.001*)* ventricular sphericity indexes. - For systolic function, there was decreased left ejection fraction *(*63 *vs*. 69; *p* < 0.001*)*, mitral ring displacement *(*4.2 *vs.* 4.7*; p* < 0.001*)*, tricuspid ring displacement *(*5.5 *vs.* 6.5; *p* < 0.001*),* mitral systolic annular peak velocity *(*6 *vs.* 6.9; *p* = 0.038*).* - For diastolic function, there was decreased mitral ventricular inflow in early diastole displacement time *(63 vs.73; p* = 0.002*)*, tricuspid ventricular inflow in early diastole deceleration time *(51 vs. 64; p* = 0.001*)*, mitral annular peak velocity in early diastole *(7 vs. 7.6; p* = 0.049*),* tricuspid annular peak velocity in early diastole *(8 vs. 8.3; p* = 0.002*)* and isovolumic relaxation time *(48 vs. 30; p* = 0.003*)*. - Neonates (1 month)—Increased DBP percentile *(71 vs. 55; p = 0.042)*, aortic mean intima-media thickness *(0.16 vs. 0.12; p* = 0.011*)*, aortic maximum intima-media thickness *(0.19 vs. 0.14; p* = 0.011*)*, carotid mean intima-media thickness *(0.07 vs. 0.06; p* = 0.035*)* and carotid maximum intima-media thickness *(0.09 vs. 0.07; P*=0.035*)* relative to body weight. - Infant (6 months)—Increased right atrial area *(2.70 vs. 2.50; p* = 0.005*)*, right wall thickness *(3.21 vs. 2.59; p* = 0.019*)*, and decreased right ventricular sphericity index *(1.82 vs. 1.91; p* = 0.010*).* - For systolic function, there was decreased left shortening fraction *(29 vs. 36; p* < 0.001*)*, mitral ring displacement *(9.4 vs. 10.8; p* < 0.001*)*, tricuspid ring displacement (*13.1 vs. 16.3*; *p* < 0.001*)* and increased heart rate *(141 vs. 132; p* = 0.002*).* - For diastolic function, there was decreased mitral ventricular inflow in early diastole displacement time *(63 vs. 66; p* = 0.014*)*, tricuspid ventricular inflow in early diastole deceleration time *(52 vs. 62; p* < 0.001*)* and increased isovolumic relaxation time *(63 vs. 50;* *p* < 0.001*).* - Increased SBP *(83 vs. 74; p <* 0.001*)*, aortic mean intima-media thickness *(1.8 vs. 1.4;* *p* < 0.001*)* and aortic maximum intima-media thickness (2.0 *vs.* 1.6; *p* < 0.001*)* relative to body weight.	[232]
Retrospective cohort study; OMEGA study, VU university Medical centre, Netherland; 1980–1995	IVF-225 Controls-225 8–18 year (mean age 12 year)	Increased SBP and DBP pressure in children conceived through IVF compared to control population at a mean age of 12.3 *(109 ± 11 vs. 105 ± 10 mmHg, p* < 0.001*; and 61 ± 7 vs. 59 ±7 mmHg,* *p* < 0.001*)*, respectively.	[233]
Cross- sectional, case-control study; IVF cases-IVF section, Department of Obstetrics and Gynaecology; University of Athens Controls-Aghai Sophia Children’s hospital; 1990–1996	IVF-106 Controls-68 4–14 year	Increased SBP and DBP standard deviation score *(0.3 vs. −0.3, p < 0.001; 0.7 vs. 0.2 p < 0.001)* in children conceived through IVF.	[237]
Clinical Trial; Swiss children IVF and Control siblings of IVF children; October 2007–April 2010	ART-65 Controls-57 Mean-11 year	Smaller flow mediated dilation of the brachial artery *(6.7 ± 1.6 vs. 8.6 ± 1.7 %; p* < 0.0001*)*, faster carotid-femoral pulse wave velocity *(7.8 ± 2.4 vs. 6.5 ± 1.3 m/s; p* < 0.001*)*, increase in carotid intima-media thickness *(410 ± 30 vs. 370 ± 20* μm*; p* < 0.0001*),* higher systolic pulmonary artery pressure *(39 ± 11 vs. 30 ± 9 mmHg; p <* 0.0001*)* in ART children compared to control children. An inverse relationship existed between pulmonary artery pressure and flow mediated dilation *(r*= −0.30, *p* = 0.001*)*.	[238]

ART, assisted reproductive technology; IVF, *in vitro* fertilization; SBP, systolic blood pressure; DBP, Diastolic blood pressure.

### 6.3. Evidence of Cardiovascular Defects from Animal Models of ARTs

Animal models of ARTs have been instrumental in providing evidence for further links between ARTs and an increased risk of cardiovascular defects (Table 6).

**Table 6:** Cardiovascular risk factors from animal models of ARTs.

Species	Age(s)	Cardiovascular Risk Factors	Reference
Sheep	61 day gestation 125 day gestation	Increased allometric growth coefficients of heart at 61and 125 days of gestation.	[163]
	125 day gestation	Increased absolute *(25.8 ± 8.9 vs. 19.0 ± 4.1, p* = 0.004*)* and relative heart weight *(6.5 ± 1.05 vs. 5.7 ± 0.76, p* = 0.022*)* in *in vitro* embryo culture group when serum was supplemented during precompaction period.	[246]
	125 day gestation	Inverse relation between heart weight and IGFR2 gene expression *(r =* −*0.675, p* < 0.001*)* and loss of methylation of IGF2R in embryo culture group when compared to control group.	[58]
	125 day gestation	A strong inverse relation between heart weight and IGFR2 gene expression in the *in vitro* embryo culture groups*(r = −0.73, p* < 0.001*).*	[247]
Cows	222 day gestation (7 months)	Increased heart girth *(56.5 ± 1.2 vs. 52.4 ± 0.9, p* = 0.01*)* and heart weight *(139.7 ± 8.3 vs. 116.2 ± 5.8, p =* 0.02*)* in embryo transfer and culture groups compared to controls.	[248]
	At birth	Increased intra-ventricular septum in *in vitro* produced groups compared to embryo transfer group (*SOF:* *11.8 ± 0.3, Co-culture: 12.0 ± 0.3, embryo transfer 10 ± 0.3, p* < 0.05) Thicker left ventricular wall during diastole in *in vitro* produced group (co-culture) when compared to embryo transfer group (14.5 *± 0.5 vs. 11.8 ± 0.6, p* < 0.05*).*	[249]
	1 year	Increased relative heart weight in embryo culture group *(4.01 ± 0.08 vs. 3.56 ± 0.12, p* < 0.02*).*	[250]
Rodents	21 days after birth	Raised SBP in males.	[62]
	12–14 weeks after birth	- Higher mean blood pressure during short-term *(p* = 0.017*)* and during chronic measurements *(p* = 0.036*)* in ART mice than in control mice. - Impaired acetylcholine-induced vasodilation in the mesenteric arteries in ART mice compared with control mice *(p* < 0.0001*)*. - Increased vascular stiffness in ART mice compared to controls measured by relationship between inner *(p* = 0.017*)* and outer diameter *(p* = 0.033*)* and carotid pressure *in vitro*. - Impaired acetylcholine-induced vasodilation *in vitro* *(p* < 0.0001*)* and increased mean arterial blood pressure *in vivo (p* < 0.001*)* was observed in the progeny of offspring of ART mate and control female.	[63,251]
	2 years after birth	Increased heart weight in embryo culture with serum compared to without serum group *(0.29 ± 0.02 vs. 0.20 ± 0.01, p* < 0.05*).*	[54]

SBP, systolic blood pressure.

#### 6.3.1. Evidence from Ruminants

Large Offspring Syndrome (LOS) is a serious side effect in culturing of sheep and cow embryos in the presence of serum [252,253] and is associated with an increase in birth weight that persists into postnatal life as well as alterations in the relative growth of organs [252,254]. Culturing sheep zygotes *in vitro* for 5 days in the presence of bovine granulosa cell layers and serum supplementation increased the allometric growth coefficients of key organs, including the heart, from as early as 61 days gestation and this change persisted to 125 days gestation [163]. Interestingly, the allometric growth coefficient of the heart was significantly increased in the fetuses that were cultured in synthetic oviductal fluid media that was supplemented with serum, despite no change in body weight at 61 days gestation. However, by 125 days gestation, the fetuses were significantly heavier with a higher allometric coefficient of the heart [163].

Sheep embryos cultured in synthetic oviductal fluid resulted in fetuses with increased heart weight compared to embryo transfer controls at 125 days gestation [246]. Furthermore, adding serum to synthetic oviductal fluid media during the first 48 h of embryonic development increased relative heart weight at 125 days gestation, which suggests that inclusion of serum during the precompaction period is more detrimental to the development of the embryo and key organs than the later stages of embryo development [246]. Young *et al*. found that a loss of methylation of insulin-like growth factor 2 receptor (IGF2R) was associated with the increased heart weight in the embryo culture groups compared to controls [58]. Interestingly, the expression of IGF2R was inversely correlated with heart weight and a stronger inverse relationship existed when the analysis was restricted to the embryo culture groups only, suggesting that IGF2R plays the role of a clearance receptor for IGF2, and thus decreased IGF2R signalling may be responsible for the increase in heart weight because less IGF2 is available to activate the IGF1R signalling pathway leading to cardiac growth [247,255,256].

Similarly, another study also reported an increased heart girth and weight in an embryo transfer and culture group at 222 days of gestation in cows (term, ~280 days) [248]. Another study in cows reported an increased intra-ventricular septum in *in vitro* produced groups (co-culture or synthetic oviductal fluid supplementation) and an increased left ventricular wall during diastole at birth in only the *in vitro* produced group (co-culture) when compared to the embryo transfer group (term, ~280 days) [249]. Few studies have investigated the impact of embryo culture and transfer on the growth and development of the heart in both fetal and postnatal life. However, one study demonstrated that despite normalisation of body weight by 1 year of age, the increase in heart weight persisted in the groups where embryos were cultured in a nutritional media for 5 days after fertilization [250].

#### 6.3.2. Evidence from Rodents

*In vitro* embryo culture during the preimplantation period resulted in increased SBP in both male and female offspring at 21 days after birth [62]. The gene expression of serum angiotensin converting enzyme, which is known to have vasoconstrictive effects through the renin-angiotensin system, was upregulated but only in female offspring [62]. Interestingly, there were no changes in the male offspring, suggesting that an alternative mechanism may be responsible for the increase in SBP [62]. Endothelial dysfunction, higher blood pressure and increased arterial stiffness were found in a mouse model of ART at 12–15 weeks after birth [63,251]. The study also found intergenerational effects of ARTs where the progeny of ART males and control female had endothelial dysfunction and higher mean blood pressure [63,251]. There was also an increase in heart weight in 20 month old mice when the embryos were cultured in media containing fetal calf serum [54].

## 7. What Are the Most Likely Mechanisms Linking ART and Risk of Cardiovascular Disease in Fetal and Adult Life?

### 7.1. Epigenetic Dysregulation

Many cardiovascular diseases are known to have an epigenetic origin [179,257]. It has been shown that individuals who were exposed to periconceptional undernutrition such as during the Dutch winter famine 6 decades ago had an altered methylation profile of imprinted and non-imprinted genes and those individuals were also found to have an increased risk of cardiovascular diseases in adulthood [258,259,260]. In animal studies, epigenetic modifications as well as increased prevalence of many cardiovascular risk factors such as increased blood pressure, adiposity and insulin resistance were found in the offspring exposed to nutritional manipulations during the periconceptional period [59,261]. These findings suggest that epigenetic dysregulation could be a plausible mechanism that links ARTs with increased risk factors for cardiovascular diseases [262].

A series of studies have identified an association between ARTs and epigenetic disorders [95,263]. Children with Beckwith-Wiedemann syndrome, which is caused by an imprinting disorder, are 3–14 times more likely to be conceived by ARTs [264,265,266,267]. Molecular analysis in these studies showed hypomethylation of maternal copy of KCNQ1 overlapping transcript 1 (KCNQ1OTI), an antisense RNA normally expressed from the paternal allele and located at one of the differentially methylated regions (DMRs) of chromosome 11p15 [264,265,266,267,268,269]. Studies have also found hypermethylation of HI9, which is located at another DMR of chromosome 11p15, as well as hypomethylation of mesoderm-specific transcript homolog protein (MEST), small nuclear ribonucleoprotein N (SNPRN), pleiomorphic adenoma gene-like 1 (PLAGL1) in ART children with Beckwith-Wiedemann syndrome [264,268,269]. There are also reports of other imprinting defects such as Angelman syndrome, Retinoblastoma, Prader–Willi syndrome and Russell–Silver syndrome in children conceived through ARTs [270,271]. At the blastocyst stage, IVF resulted in aberrant H19 methylation in humans and mice [226,272]. Large offspring syndrome LOS is a frequent occurrence in livestock conceived through ARTs and is also caused by loss of methylation in the maternal DMR of IGF2R, which is an imprinted gene [58].

A study investigating the DNA methylation levels of more than 700 genes (1536 CpG sites) in the placenta and cord blood reported lower mean methylation at CpG sites in the placenta and higher mean methylation at CpG sites in cord blood from 10 children conceived *in vitro* compared to 13 children conceived naturally. Upon examining the gene expression levels of a subset of genes that had altered methylation levels, CCAAT/enhancer-binding protein alpha (CEBPA) in cord blood and CEBPA, MEST, neuronatin (NNAT) and serpin peptidase inhibitor, clade F (alpha-2 antiplasmin, pigment epithelium derived factor), member 1(SERPINF1) in placenta had significant difference in mean transcript levels [273]. Interestingly these genes are known to be associated with adipocyte development and differentiation, insulin signalling and/or obesity [274,275,276,277].

Increased methylation of the imprinted genes H19, glucosyltransferase 2 (GTL2) and decreased methylation of paternally-expressed gene 3 (PEG3) were found in the aorta, but not in the liver of the mice conceived through ART [251]. This alteration of the methylation levels was transmitted to the next generation with levels comparable to the methylation of the parent generation [251]. Interestingly, the altered methylation levels were restored in ART mice and in its progeny with the administration of butyrate (a histone deacetylase inhibitor) [251]. As previously mentioned, the study also found vascular dysfunction and arterial hypertension in ARTs mice and its offspring. Consistent with this finding, the study reported an increased DNA methylation of the promoter of the eNOS gene in the aorta of the ARTs mice, which is an important regulator for systemic vascular function [251]. This increased methylation level resulted in decreased eNOS mRNA expression in the vasculature and lower levels of nitric oxide in the plasma of ARTs mice [251]. In addition, butyrate administration to ARTs mice also normalized DNA methylation of the promoter of the eNOS gene, eNOS mRNA expression and plasma nitric oxide concentration [251].

**Figure 3:**
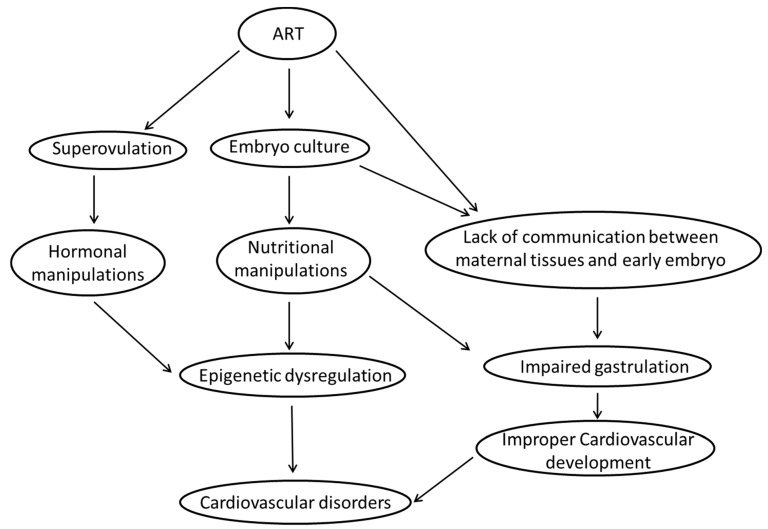
Diagrammatic representation of the possible links between ARTs and cardiovascular disorders [284,293,297].

These altered methylation levels provide strong evidence for epigenetic dysregulation as a result of ARTs, which could possibly affect the expression levels of genes involved in cardiovascular regulation as well as other major regulatory pathways increasing the risk of long-term diseases in adult life [63]. These alterations in epigenetic mechanisms may stem from several steps involved in ARTs such as ovarian stimulations and *in vitro* embryo culture (Figure 3). Studies have investigated the impact of these steps separately, which may underpin the source for epigenetic dysregulations [163,278,279].

#### 7.1.1. Ovarian Hyperstimulation/Superovulation

Superovulation occurs during meiosis of oocyte development/maturation when the oocytes are still acquiring imprinting marks and may lead to imprinting defects. In humans, studies have reported hypomethylation of the KCNQ1OT1 DMR (KvDMR1) in the germinal vesicle and metaphase I stage as well as hypomethylation of KCNQ1OT1 in the metaphase II stage of the superovulated oocytes, which suggests that ovarian hyperstimulation may result in the release of immature or young oocytes that have not undergone complete methylation, including the establishment of appropriate imprinting [85,280]. Imprinting errors have been found in H19 and MEST in metaphase II oocytes after ovarian stimulation, however, it could not be determined whether alterations in the methylation patterns of these genes were associated with the superovulation process itself or the age and infertility status of the patients [281]. There is evidence that superovulation can affect the quality of the embryo as well as the uterine milieu, and these factors can indirectly affect the epigenetic status of the oocyte [48].

In animal studies where infertility is not a confounding factor, an abnormal methylation pattern at the 2 cell stage was found in the embryos from superovulated mice using immunofluorescent staining [282]. Downregulation of candidate reprogramming genes which are involved in base excision repair proteins such as APEX nuclease (multifunctional DNA repair enzyme) 1 (APEX1), polymerase (DNA directed), beta (POLB) and the 5-methyl-CpG were found at the morula stage of the mouse embryo after superovulation and suggests that superovulation may also hamper the maintenance of imprinting [278]. In line with this finding, lower protein expression levels of APEXI were observed in both early and late morula stages and the level of protein expression correlated the mRNA expression levels [278]. Loss of methylation of SNRPN, KCNQ1OT1 and PEG3 in the blastocyst stage was also observed in a dose-dependent manner with higher doses of hormones resulting in greater imprinting disturbances [283]. Superovulation in mice decreased the methylation levels of H19 and significantly increased the methylation levels of PEG1 and SNRPN imprinted genes in the sperm of the offspring in both the first and second generation [53]. Together these data suggest that changes in global methylation may account for spontaneous embryo loss and developmental failure, while locus-specific epigenetic errors may result in defined phenotypes associated with genomic imprinting disorders; thereby suggesting that oocytes from superovulated animals may have a reduced ability to complete the global reprogramming necessary for proper development [282,284].

#### 7.1.2. *In Vitro* Embryo Culture

*In vitro* embryo culture occurs during the sensitive period of maternal and paternal demethylation and remethylation and thus may affect the maintenance of genomic imprinting during this process [285]. There is evidence of abnormal methylation, mainly due to embryo culture, as a result of the IVF procedure in mice [286]. For example, embryo culture of preimplantation mouse embryos using Whitten medium resulted in biallelic expression and loss of methylation of the H19 gene [287]. Similarly a greater loss of methylation was observed in H19 after embryo culture in M16 medium than in G1/G2 medium [287]. There were also differences in the gene expression of 114 genes in the preimplantation embryos of the mice cultured in Whitten culture medium and 29 genes when cultured in KSOM + AA [61]. Biallelic expression of the imprinted gene H19 was found in the placenta which persisted till mid gestation when the embryos were cultured in whitten or KSOM + AA medium [288]. There is also evidence of perturbed gene expression after embryo culture [163]. Increased expression of the imprinted genes, IGF2 and H19 and growth factor receptor binding protein 10 (GRB10), along with increased methylation of H19 and decreased methylation of growth factor receptor binding protein 7 (GRB7) were observed in fetuses that had undergone preimplantation embryo culture in M16 medium with fetal calf serum supplementation [61]. *In vitro* culture with serum supplementation also resulted in hypomethylation of IGF2R associated with LOS in ruminants [58]. It has been speculated that culture media procedures may induce abnormal methylation at imprinted loci by facilitation the removal of methyl groups from cytosine bases or interfering in normal gamete development which may lead to incomplete imprint erasure and/or reestablishment of methylation pattern [95,285]. The methionine content of the media may also affect DNA methylation and imprinting [102,289].

These findings provide substantial evidence that *in vitro* embryo culture can impair epigenetic mechanisms. Studies investigating epigenetic dysregulation associated with both ovarian stimulation and embryo culture found that defects in imprinting were neither observed in all the embryos nor at every imprinting locus. Some loci were affected more than others. Hence, more studies are required to understand the stochastic nature of these imprinting defects.

### 7.2. Impaired Gastrulation

Early interaction between the embryo and the endometrium is critical for proper development and implantation of the embryo [290,291]. This in turn determines proper gastrulation and organogenesis. Embryo cultured in media *in vitro* lacks these early interactions and the exchange of factors between embryo and endometrium. This can alter the timing of expression of Hox genes, which control the body plan of an embryo along the anterior-posterior (head-tail) axis [292,293]. In addition, studies have shown that culture of the embryo *in vitro* affects the proper differentiation of the inner cell mass, which are precursors of ectoderm and endoderm (germ layers formed during gastrulation) [294,295]. These two, individually or in combination, can alter gastrulation and subsequently organogenesis. It is reasonable to speculate that organogenesis of the heart, which is the first organ to form, may be affected by procedures involved in ARTs and abnormalities in heart development are known to cause congenital defects and other cardiovascular disorders (Figure 3) [296].

## 8. Conclusions

This review summarises the literature showing that ARTs are associated with an increased risk of cardiovascular disease. Many studies have been conducted that have shown an association between ARTs and congenital heart defects. It is clear from the available evidence that the association is not restricted to a specific type of ARTs. However, a major gap of literature exists in terms of our understanding of the long-term effects of ARTs in childhood and adulthood. Some follow-up studies have confirmed these findings in children of ARTs in the fetal and neonatal periods. Given the young age of the ART population, it is necessary to perform life course studies that will provide valuable evidence not only to ensure better medical diagnostics and care for these children but also to call for more studies to pinpoint the mechanistic alterations that are responsible for ARTs induced cardiovascular risk factors. Understanding the important events that occur during the developmental periods when ARTs procedures are applied have identified mechanisms underlying the link between ART and an increased risk of cardiovascular defects in the embryonic period and cardiovascular disease in postnatal life.

Epigenetic alterations have emerged as a key mechanistic alteration that can predispose an increased risk of cardiovascular disorders in the ART population. A major challenge is to isolate the effects of infertility of the parents that sought ARTs procedures and may render epigenetic alterations in the offspring. In this regard, many animal models of ARTs have been a major source of evidence suggesting the effects of ARTs procedures on cardiovascular dysfunctions and epigenetic alterations. This warrants further studies to pinpoint specific epigenetic alterations that directly link to the development of cardiovascular disease in later life. Consequently, a genome-wide epigenetic profiling of children conceived through ARTs during the embryonic stages can be used as a tool to identify embryos that carry epigenetic alterations linked to the development of cardiovascular disease in adult life. Furthermore, epigenetic profiling can also be conducted in both neonatal and adult life, to identify individuals with a higher risk of developing cardiovascular disease in later life.
